# Supplementary material for: Are carbon emissions trading and green financial instruments synergistic? -Comprehensive quantitative research based on content analysis
Source: PLoS One. 2024 Mar 7;19(3):e0298601. doi: 10.1371/journal.pone.0298601 (PMC10919601; doi:10.1371/journal.pone.0298601)
Supplement: S1 Data — (ZIP) [file pone.0298601.s001.zip › Data/地级市Z_F工作报告.zip!/╕ú╜¿/╒─╓▌╩╨/└┤╫╘╣1⁄2╓┌║┼í╛┬φ┐╦╩2╛▌═°í┐_2012.docx]

各位代表：

　　现在，我代表漳州市第十四届人民政府向大会作报告，请予审议，并请市政协委员和列席人员提出意见。

　　一、过去五年的工作

　　2011年是本届政府任期最后一年，也是“十二五”开局之年。一年来，在省委、省政府和市委的正确领导下，在市人大的法律、工作监督和市政协的民主监督下，全市上下深入贯彻科学发展观，全面完成了市人大确定的各项目标任务。全市地区生产总值1768.2亿元，增长14.7%；财政总收入174.5亿元，增长25.2%，其中地方级财政收入112.1亿元，增长26.6%；全社会固定资产投资1115.7亿元，增长38.4%；农业总产值519.9亿元，增长4.6%；规模工业增加值737亿元，增长22%，规模工业总产值2617.3亿元，增长21.1%；外贸出口64.9亿美元，增长28%；实际利用外资8.9亿美元，增长26.6%；社会消费品零售总额529.4亿元，增长19.2%；居民消费价格总水平上涨5%；城镇居民人均可支配收入21137元，增长14.4%；农民人均纯收入9128元，增长16.1%；城镇登记失业率2.02%；人口自然增长率6.22‰;单位生产总值能耗下降3.3%，顺利完成省下达的节能减排任务。

　　一年来的主要工作和成效是：

　　（一）“五大战役”实现新突破。全面掀起“再干150天、实现五大战役新突破”热潮，重点项目战役投资667.5亿元，完成计划131.8%，列入省级的54个在建重点项目投资297.7亿元，完成计划168.3%；新增长区域战役投资427.7亿元，完成计划126.6%；城市建设战役投资142.3亿元，完成计划161%；小城镇改革发展战役投资294.9亿元，完成计划114.2%；民生战役投资228.3亿元，完成计划136.2%。古雷石化启动项目基本完工，今年上半年即将试投产；福欣特钢进入全面建设阶段，年内可竣工投产；旗滨玻璃第四条生产线建成投产，第五、第六条生产线开工建设；28个投资10亿元以上的重大生产性项目加快建设，将在今明两年建成或投产；古雷炼化一体化、漳州军民合用机场、长泰枋洋水利枢纽工程、市区第二饮用水源等一批重特大项目前期工作取得实质性进展。

　　（二）“三维”招商凸显新成效。坚持“宁可少一点也要好一点，宁可少一点也要实一点”，有力有效推进央企、外企、民企“三维”招商工作。成功举办上海、福州民营企业产业项目洽谈会，招商成果位居全省第二。全市共对接央企项目12个，总投资468.5亿元；新批外资项目149个，注册合同外资12.6亿美元；对接民企项目88个，总投资2239亿元。常山开发区成为全省首个国务院侨办引智引资重点联系单位。“农博会•花博会”展期从5天延长至3个月，规模更大、特色更浓、水平更高，预计游客可达百万人次；已有1172家国内外采购商前来洽谈订货，达成订货协议金额超过29亿元；签约“三维”项目87个、总投资373亿元，“农博会•花博会”作为对外经贸合作的平台作用日益凸显。

　　（三）结构调整取得新进展。现代农业加速提效。新增4个国家级农业龙头企业。农产品出口超30亿美元，增长50%以上，其中水产品出口增幅超过70%。新增绿色食品、有机食品12个、无公害农（水）产品认证48个。成为南方首个获得“中国食品名城”称号的城市。粮食总产量72.1万吨，完成造林91万亩。工业经济扩量提质。实施工业“千百工程”项目1200个，投资突破500亿元，500个项目建成投产。新增亿元企业128家，总数达到500家。高新技术产业快速发展。新认定高新技术企业51家，高新技术产业产值610亿元，增长30%。新增2个国家高新技术产业化基地，被列为国家“十城万盏”试点示范城市。漳州市和芗城、龙文、龙海、南靖分别荣获“2011年全国科技进步先进市、县（区）”荣誉称号。品牌战略成效显著。新增中国驰名商标4个，国家地理标志商标10个。第三产业加快发展。一批城市综合体和物流项目相继投入使用。“万村千乡”市场工程深入实施，建成14个农村集贸市场、2个日用消费品和农资配送中心、180家“农家店”。金融业发展迈出坚实的步伐。泉州银行、招商银行入驻，漳州市第一家农村商业银行开业，漳州海峡金融交易所在北京挂牌。旅游业升温提速。旅游总收入120亿元，南靖土楼旅游景区被评为国家5A级景区，华安仙都镇和长泰山重村分别被评为全国特色景观旅游名镇和名村。

　　（四）服务发展推出新举措。出台《关于扶持企业发展的若干意见》、《漳州市银行业金融机构支持经济发展考核奖励办法》和《关于促进当前工业稳定增长六项措施》及其补充措施等“暖冬”政策，深入开展“帮企业渡难关、帮项目破难题”活动和以“六送”为主题的“暖冬”行动，推动项目建设提速增效。全市各项贷款余额1008亿元，增长20.6%，增幅居全省前列。6家企业成功上市，募集资金69.3亿元；20个项目BT融资75.5亿元；发行中期票据、企业债券、城投债券22亿元。深化行政审批制度改革，精简下放145项行政审批项目。出台《2011年度漳州市“五大战役”项目审批服务考评细则》和《关于进一步深化行政服务工作的十四条意见》，推动行政审批效率和服务水平同步提高。

　　（五）民生改善迈上新台阶。32件为民办实事项目累计投资超60亿元，完成计划近150%。全市1.7亿元超收财力主要用于民生事业。开工建设各类保障性住房26921套，超额完成省下达计划。校安工程开工面积68.5万平方米，新改扩建40所公办幼儿园。“造福工程”搬迁人数5945人。新增4项国家级非物质文化遗产。新改扩建乡镇综合文化站23个、农家书屋350家。医疗卫生体制改革扎实推进，新农合参合率达99.7%，位居全省第一。新农保实现全覆盖，全面完成全市400万张社保卡发放任务。新增城镇就业5.9万人。出台《关于进一步加强城镇弱势群体保障工作的实施意见》，市慈善总会正式成立。制订实施稳定物价十二条措施，切实加强物价监控和物资调度。全面开展专项整治，有效保障食品、药品安全，制售假冒卷烟现象得到有效遏制。深入实施“安全生产年”活动，安全生产四项控制指标全面下降。人口计生工作有效提升，实现优质服务县全覆盖，被确定为“全国婚育新风进万家活动”示范市、“全国人口计生综合改革”示范市和“全国创建幸福家庭活动”试点市，综合评估位居全省第一。

　　加强和创新社会管理，制定出台《漳州市人民政府领导信访件办理办法》，认真开展“走基层、大接访”活动。实施依法处理信访事项“路线图”，完善“三位一体”大调解工作格局，建立健全维稳“三支队伍”，推广和谐征迁工作法，社会和谐稳定。

　　（六）城乡建设呈现新面貌。厦漳同城深入推进。《厦漳泉大都市区同城合作框架协议》正式签署并全面实施。成功举办厦漳同城化产业对接恳谈会，现场对接项目68个，总投资超300亿元。厦漳跨海大桥等重大基础设施项目加快建设。以规划引领提升中心城市建设和管理水平。邀请国内外知名规划设计专家莅漳指导，修编完善《漳州市中心城市总体规划》，提升城乡规划水平。投入近70亿元建设城市道路、桥梁等基础设施。江滨公园建成投入使用，蒲姜岭生活垃圾焚烧发电厂、内河引水工程、芝山公园开工建设。碧湖生态园建设加快推进，圆山新城开发正式启动。全国文明城市创建活动有序开展，荣获“全国文明城市”提名资格。省级“森林城市”通过验收。“城乡环境百日整治”行动成效明显，城市运行更加规范有序，面貌更加清爽整洁。县域经济竞相发展。龙海市在全国县域百强县的位次进一步前移。多个县市进入全省县域发展“十强”、“十佳”、“十优”。新农村示范村建设和“四位一体”村容整洁行动深入推进，城乡人居环境进一步改善。

　　（七）政府建设得到新加强。自觉接受人大及其常委会监督，依法执行人大决定决议，认真办理人大常委会审议意见及人大代表建议、批评和意见，积极配合人大常委会开展执法检查、人大代表视察及专题调研。支持人民政协履行政治协商、民主监督、参政议政职能，认真办理政协提案议案，积极采纳委员调研成果。全年共办理市人大代表建议145件、市政协提案275件，办复率均为100%。主动听取各民主党派、工商联、各人民团体、无党派人士和社会各界人士的意见。扎实推进政府机构改革，市县两级政府工作机构数分别精简18.2%和20.5%。严格执行《市政府工作规则》，全面落实政府信息公开制度，改进行政复议工作，深化机关效能建设，完善政府绩效评估，推进政府工作规范化、公开化、高效化。严格执行党风廉政建设责任制，认真落实《党员领导干部廉洁从政若干准则》，出台《漳州市政府公务活动接受赠品管理规定》。强化行政监察、审计监督和民主评议工作，坚决纠正损害群众利益的不正之风，政风行风进一步好转。

各位代表：

　　过去的五年，我们面对复杂多变的国内外形势，成功应对国际金融危机，有效抵御多次特大自然灾害，有力推动经济提速发展，为实现跨越发展奠定了坚实的基础。发展实力大幅提升。2011年全市地区生产总值、人均地区生产总值均比2006年翻一番以上，财政总收入是2006年的2.7倍。古雷被列为全国重要临港石化产业基地和台湾石化产业园区，漳州开发区升格为国家级经济技术开发区。发展方式加快转变。2011年规模工业总产值和规模工业增加值均是2006年的3.4倍，三次产业比例从2006年的21.6∶41.8∶36.6调整为16.7∶49.2∶34.1；节能减排指标全面完成，市区、县城污水处理率分别达到88%和79.9%、垃圾处理率分别达到99.3%和99.4%。森林覆盖率保持全省前列，通过“国家园林城市”复核验收。发展后劲显著增强。古雷石化、旗滨玻璃等一批重大工业项目相继投建投产，基础设施全面改善，厦漳跨海大桥进展顺利，新建高速铁路里程226公里、高速公路里程289公里，新增农村水泥路2860公里、港口吞吐能力2000万吨。2011年全社会固定资产投资是2006年的4.6倍。发展成果普惠于民。城镇居民人均可支配收入、农民人均纯收入分别从12511元、5075元提高到21137元、9128元。全面实现城乡免费义务教育，文化、体育事业取得长足进步，南靖、华安土楼申遗成功。医药卫生体制改革全面推进，荣获“国家卫生城市”称号。社会保障体系不断完善，人民群众从发展中得到更多实惠，生活水平进一步提高。

　　我们还科学编制了“十二五”规划，顺利完成第六次全国人口普查。与此同时，妇女儿童、老干老龄、老区建设、民族宗教、外事侨务、新闻出版、广播电视、地方志、档案、地震、气象、残联、红十字会、国防动员、支前双拥、海防人防、应急管理等工作也都取得了新成效。

　　回顾本届政府的工作，我们深切体会到：必须始终坚持科学发展，加快转变促跨越；必须始终坚持解放思想，先行先试促跨越；必须始终坚持执政为民，改善民生促跨越；必须始终坚持生态引领，保护环境促跨越；必须始终坚持真抓实干，勇担责任促跨越；必须始终坚持市委领导，凝心聚力促跨越。

　　各位代表，五年成绩来之不易。这是省委、省政府和市委正确领导的结果，是全市人民齐心协力、团结奋斗和各方面共同努力的结果。在此，我代表市人民政府向为漳州发展付出辛勤劳动的全市人民，向给予政府工作大力支持、有效监督的人大代表、政协委员、各民主党派、工商联、各人民团体、无党派人士、离退休老同志和社会各界人士，向积极服务漳州发展的中央、省驻漳机构、驻漳部队、武警官兵、公安民警，向关心支持漳州发展的港澳台同胞、海外侨胞和国际友人，表示崇高的敬意和诚挚的感谢！

　　在看到成绩的同时，我们也清醒地认识到，漳州的发展还面临不少的困难和不足：经济规模总量偏小，产业结构不尽合理，综合实力与先进地市差距较大；中心城市辐射带动能力不强，功能配套不够完善，城市品位有待提升，城镇化进程亟需进一步加快；农村农业基础设施仍然较为脆弱，农民持续增收难度较大；关系民生的社会事业欠账较多，物价特别是食品类价格仍然高位运行，生态环境保护压力加大；影响社会稳定的各种因素仍然存在，安全生产、社会治安存在薄弱环节，社会管理创新有待加强；政府职能需要进一步转变，一些工作人员服务意识不强、破解难题办法不多，一些领域腐败现象还时有发生。对这些问题，我们一定要高度重视，认真加以解决。

　　二、今后五年的目标任务

　　今后五年，是漳州加快发展、跨越发展的重要战略机遇期，也是漳州大项目加快落地、大产业加快集聚、城市化加快推进的重要时期。我们要敢于创新观念，勇于创造优势，善于创建平台，扎实推进“三化”并举、“三群”联动、“三维”招商，朝着“更加优美、更加和谐、更加幸福”的方向奋力拼搏。

　　按照省第九次党代会和市第十次党代会的部署，今后五年漳州市政府工作的总体要求是：高举中国特色社会主义伟大旗帜，以邓小平理论和“三个代表”重要思想为指导，全面贯彻落实科学发展观，紧紧围绕“海西建设、漳州先行”，继续实施“依港立市、工业强市、开放活市、科教兴市”发展战略，突出“工业化提速、城市化提升、现代农业提效”发展重点，坚持以增量促调整，以转变谋跨越，以规划为引领，以对台求先行，以生态创优势，以文化强实力，以惠民增和谐，努力在科学发展的轨道上实现新的更大跨越，在更高起点上建设创业创新的活力漳州、又富又美的幸福漳州。

　　今后五年的奋斗目标是：经济发展速度快于全省平均水平，至2016年，地区生产总值超过3500亿元，财政总收入超过480亿元，区域竞争力显著提升，经济综合实力进入全省前列，在全国地级城市综合竞争力排位力争进入50强；公民思想道德、科学文化和健康素质显著提高，民主法制建设更加健全，基本公共服务体系覆盖城乡；生态文明建设和生态环境质量继续保持全国、全省前列。

根据以上总体要求和奋斗目标，今后五年的主要任务是：

　　（一）以增量促调整，推动固定资产投资持续增长。增强投资的主导地位和引领作用，把产业结构升级、临港工业集聚、基础设施建设、城乡统筹发展、民生福祉改善、生态环境保护作为投资重点，保持固定资产投资持续快速扩张。集中力量加快建设事关发展、惠及民生、展示形象的“双十”工程。积极推进大型码头群、高速铁路、高速公路、城际轨道、同城大道、沿海大通道、海底隧道、军民合用机场等基础设施建设，打造高效便捷的集疏运体系。策划、储备、实施一批重大生产性、基础性项目，加快一批重大项目前期工作，为跨越发展提供强劲支撑和持久动力。

　　（二）以转变谋跨越，推动产业发展层次加速跃升。加强港口规划建设，积极参与厦门东南国际航运中心建设，加快石油化工、特殊钢铁、装备制造等临港产业发展，着力打造全国重要临港石化产业基地、海西先进制造业基地。以良种、加工、营销为重点，更加注重用工业化理念发展农业，扎实推进农业生产经营精准化、标准化、规模化，实现从“鱼米之乡”到“国人厨房”和“世界餐桌”的跨越。着力推动金融保险、商贸物流、休闲旅游、文化创意、总部经济等服务业成为支柱产业，构建与产业发展相协调、居民消费升级相适应的现代服务业体系，三次产业比例力争调整为10∶52∶38。

　　（三）以规划为引领，推动城市辐射能力不断扩大。按照“田园都市、生态之城”的发展定位，围绕“水城、绿城、历史文化名城”的建设目标，以国际化视野和前瞻性眼光规划城市，科学设计城市天际线、轮廓线、景观线、交通线。积极推进厦漳泉同城化，加快实施“中心东移、跨江南扩、面海拓展”城市发展战略，打造“一中心六组团”的大漳州发展格局。统筹旧城改造与新区开发，实施“城市建设五年行动计划”，深入开展“十大工程”建设，完成“两个千亿元”投资目标，实现城市建设上台阶、内涵上品位、面貌大变样。健全完善城市精细化管理长效机制,积极争创全国文明城市，营造整洁卫生、规范有序、温馨宜居的城市环境。

　　（四）以对台求先行，推动改革开放领域纵深发展。突出对台优势，着力先行先试，全面深化漳台交流合作。规划建设好台商投资区，以引办台资项目为抓手，努力把漳州建成两岸交流合作引领区、对接台湾产业密集区。强化“三维”招商，形成内外联动、互利共赢的开放型经济体系。扎实推进外贸增长方式转变，不断提高外贸整体实力。加快实施“走出去”战略，支持企业境外投资。深化行政审批制度改革、农村综合改革、国有企业改革、事业单位分类改革和资源要素配置市场化改革，率先建立充满活力、富有效率、更加开放、有利于科学发展的体制机制。

　　（五）以生态创优势，推动“两型社会”建设有效突破。积极创建国家生态城市和森林城市，强化能源资源节约和生态环境保护，加快建设资源节约型、环境友好型社会。大力发展低碳产业和循环经济，强力推进节能减排，坚决淘汰落后产能，确保全市主要污染物排放总量削减率不低于全省平均水平。深化城乡环境综合整治，实现九龙江等重点流域和城乡环境质量显著改善。强化饮用水源保护，加快市区第二饮用水源建设步伐，切实保障水环境安全。

　　（六）以文化强实力，推动文化大发展大繁荣。全面实施文化“八大工程”。坚持把社会主义核心价值体系建设融入国民教育、精神文明建设全过程，积极推进文化改革创新，努力营造崇尚创业创新、开放开明的社会风气。加快实施重点文化惠民工程，构建覆盖城乡的公共文化服务体系，发展具有地方特色的文化创意产业，推动文化产业成为支柱性产业。深入研究和挖掘具有漳州特色的闽南文化，积极推动“海上丝绸之路”申遗工作，促进非物质文化遗产、民俗民间文化和历史文化名城、名镇、名村的保护开发。

（七）以惠民增和谐，推动群众幸福感持续增强。加强和创新社会管理，着力解决满足群众生存需求、发展诉求、幸福追求的“三件要事”，切实在化解社会矛盾、加强公共服务、促进公平正义、创新机制方法、夯实基层基础五个方面下功夫，提高社会管理科学化水平。坚持把新增财力、公共资源更多向民生事业倾斜，尽力缩小城乡差距、贫富差距，加速社会保障普惠化、公共服务均等化、人居环境生态化，努力实现学有优教、劳有多得、病有良医、老有善养、住有宜居。

　　三、扎实做好2012年的工作

　　2012年是党的十八大召开的喜庆之年，也是全面落实省第九次党代会、市第十次党代会战略部署的开局之年。按照“稳中求进”、“好中求快”的总基调，结合漳州实际，以好促稳，以快求进，立足快、追求好。全年主要预期目标为：地区生产总值突破2000亿元，增长14%；财政总收入突破200亿元，增长20%，其中地方级财政收入增长20%；全社会固定资产投资增长25%；规模工业增加值增长21%，规模工业总产值增长20.7%；农业总产值增长4.2%；外贸出口增长15%，实际利用外资增长20%；城镇登记失业率控制在4%以内；社会消费品零售总额增长17%，居民消费价格总水平涨幅控制在4%左右；人口自然增长率控制在7.1‰以内；城镇居民人均可支配收入和农民人均纯收入增长14%；完成省下达的节能减排任务。

实现上述目标，必须着重做好以下九个方面的工作：

　　（一）多措并举做大做强实体经济

　　突破重点区域，促进产业集聚。把古雷台湾石化产业园区开发建设作为全市工作重中之重，着力推动古雷石化启动项目上半年投产，抓紧推进炼化一体化、中石油百万吨级乙烯项目前期工作，开工建设二甲苯、间苯二甲酸等7个石化项目，加快30万吨级码头、引水工程、热电厂、变电站等基础设施建设，全年实现固定资产投资超75亿元，工业产值超200亿元，财政总收入超10亿元。台商投资区要着力提升承载能力，加快福欣特钢、联盛纸业、圣莉雅壁纸等重大项目建设，确保年内如期投产，全年力争实现工业产值500亿元以上，财政收入22亿元以上。南太武滨海新区要加快构筑以沿海大通道为重点的基础设施体系，着力推进正新轮胎、首钢-凯西钢铁、漳浦滨海旅游区、白塘湾国际旅游度假城等重大项目实施，尽快建成重要的新经济增长极，力争全年投资75亿元以上。九龙生态经济区要做大规模，提升效益，扎实推进芗城联想新型科技中心、华润金峰城市综合体、华安“义大世界”等项目建设，力争全年投资超34亿元。其他14个省级以上开发区要确保固定资产投资、工业增加值、财政收入均增长20%以上。

　　加速扩量提质，增强产业支撑。突出“抓龙头、铸链条”，加快98个省级、300个市级在建重点项目和38个省级预备重点项目建设进度，确保古雷翔鹭石化、福欣特钢、联盛纸业、旗滨玻璃第5-8条生产线4个投资超百亿元项目和康师傅饮品、汉晶光电、万利太阳能、英博雪津啤酒等28个投资超10亿元项目竣工投产，开工建设1000家规模工业企业项目，力争全年实现工业投资600亿元以上，新增工业产值1000亿元以上。完善支持中小企业发展、促进工业稳定增长等政策措施，健全中小企业融资担保风险补助和小企业贷款风险补偿机制，落实信贷倾斜和税收优惠政策，进一步清理涉企收费，积极帮助企业开拓市场、协调用工、改善管理、扩量提质，壮大规模企业群体。加强电网建设，保障企业用电需求。大力发展海洋经济，推进港航、铁路、公路等重大基础设施项目建设，构建一体化集疏运体系。高标准谋划一批船舶与海洋工程装备、海洋生物、海洋运输和港口物流项目，加快形成以厦门湾、东山湾为依托的海洋经济密集区。

　　促进消费扩张，壮大第三产业。出台扶持政策，优化服务业发展环境。围绕建设区域性物流中心的目标，培育引进物流市场主体，建设一批大型商贸物流项目和物流园区，大力发展“总部经济”、“流量经济”、“道口经济”。加快培育更多中心商业圈，规划建设一批特色商业街区。制定鼓励政策，为建筑企业、运输企业入驻创造良好环境。壮大金融服务业，引进一批金融机构，支持非银行金融机构发展。推进智慧城市建设，加速信息服务业成长。加快旅游产业发展，进一步整合文化、滨海、温泉资源，推进土楼旅游区、滨海旅游区和温泉养生区等建设；充分发挥农业生态、田园风光优势，大力发展农家乐、休闲游等乡村旅游。推动文化与旅游产业融合，加快星级酒店、文化休闲场所、特色购物等旅游设施配套，延伸旅游产业链，推动旅游产品由观光游览型向休闲度假型转化、旅游市场由旅游客源地向旅游目的地转化。鼓励旅游、健身、文化等消费，积极发展网络购物等新兴消费业态，支持发展消费信贷。

　　着力自主创新，提升产业档次。积极打造创新平台，抓紧申报以南靖高新区为核心的国家级漳州高新技术开发区，加快节能照明、智能家电2个国家级高新技术产业化基地建设，争创“国家科技进步先进市”。提升“6•18”科技成果对接转化能力，扎实推进“蓝火计划”，完善产学研等创新机制。深入实施人才强市战略，积极引进科技领军人才和一线创新人才，壮大一批自主创新型科技示范企业。争取新认定高新技术企业30家以上，高新技术产业产值达700亿元以上。深入实施名牌发展战略，力争新增驰名商标4个、著名商标50个。

　　（二）强化措施加速现代农业提效

　　用工业理念发展农业。制定扶持重点农产品、水产品加工和农业龙头企业的政策措施，推动农业特色产业集群加快发展。以“中国食品名城”为平台，垂直整合产业链，提升农业全产业链配套程度，推动一产向二、三产业转化。修订完善《食品工业发展规划》，建设10个食品加工示范区，力争产值上亿元企业超100家、上5亿元企业超25家、上10亿元企业超10家。把食品文化创意纳入文化强市发展战略，与会展、旅游、物流等新兴产业联动，与电视、出版、演艺、动漫等强势产业联盟，推动食品产业做大做强。

　　用现代科学技术提升农业。编制现代种业发展规划，实施种业创新与产业化工程，着力建立现代种业体系。深化漳台农业合作，拓展台湾农民创业园，扩大两岸农业技术交流。推广优良品种，完成粮食种植面积179万亩。大力发展钢质渔船，支持发展远洋捕捞。加快“一个园区、三个中心”建设，打造优质种子种苗引进试验、示范推广基地。扎实推进农业“五新”进村入户，大力培养农业科技人才，积极引进关键技术，提升农业技术含量。

　　用现代营销方式拓展农业。以海峡两岸（福建东山）水产品加工集散基地、海峡（福建漳州）花卉集散中心、闽荷花卉交流合作中心等为依托，推动“农博会•花博会”常态化经营，构建具有产品集散、仓储运输、加工配送等多功能的网络化物流配送体系。推动农超对接、农产品协议流通以及农产品电子商务，发展“订单农业”、“合同农业”，构建农产品流通体系。利用全国绿色食品单列管理市的政策优势，向外拓展标准化生产基地、出口备案基地，逐步形成以漳州为总部、全国为依托的新布局。

　　用现代基础设施保障农业。落实强农惠农富农政策。实施152个水利工程项目，重点加快枋洋水利枢纽、古雷区域引水、九龙江防洪、龙海九九坑水库等工程进度。推进农村路网、信息化和农村电网改造工程。开展土地整理，建设标准农田。大力发展设施农业，建设一批农业产业化示范区和花卉产业园区。抓好“菜篮子”工程，加大城市副食品基地建设和管理力度。高度重视农产品质量安全，发展“三品一标”，创建一批全国绿色食品基地县。抓好重大动植物疫病防控，健全农产品质量安全监管和可追溯体系。

　　（三）全力以赴推动“五大战役”再突破

　　拓展战役的领域和内涵。把战役实施作为推动“三化”并举、“三群”联动的有力抓手，作为拉动内需、发展实体经济的有效途径，作为保障和改善民生的重要载体，持之以恒打好“五大战役”。进一步拓展战役领域，把城乡规划、农业产业化、农林水利、“四绿”工程、城镇美化、造福工程、饮水安全、减灾防灾、旅游等领域项目纳入战役范围。初步安排，重点项目建设战役实施项目300个，年度投资574.5亿元，力争开工建设10个投资超百亿元、100个投资超十亿元项目。加快3条铁路建设进度，确保龙厦铁路上半年通车运营；续建、新建10个高速公路项目，开工建设新江东大桥。新增长区域发展战役实施项目368个，年度投资416.8亿元。城市建设战役实施项目523个，年度投资230亿元。小城镇改革发展战役实施项目342个，年度投资300亿元。民生工程战役实施项目102个，年度投资205亿元。

　　提升项目的层次和质量。致力招大商、选好商，瞄准世界500强、央属企业、大型民企、台湾双百企业、华侨华人大财团，主动对接衔接，力求招引重大项目实现新突破，力争全市引进“三维”项目200个以上，总投资超3500亿元。发挥亲情乡谊优势，重视以台引外、以侨引外，实现引进世界500强新突破。完善项目动态跟踪系统，强化抓在建、促履约、推在谈，提高项目履约率和开工率，推动已签约的149个外资项目、26个央企项目、58个民企项目加快落地建设。

　　坚持战役的思维和方法。注重“全程跟踪、全程服务、全程协调”，强化“要素保障、服务保障、纪律保障”，推进“五大战役”更好更快实施。进一步优化投资环境，健全跟踪督查考核机制，持续深化“两帮”活动和“暖冬”行动，推动帮扶工作常态化、制度化、责任化，促进项目加速投建、企业加快投产。

　　（四）用心用情用力办好民生事业

　　增加城乡居民收入。积极拓宽就业渠道，重点帮扶大中专毕业生、农民工、零就业家庭、退役军人等群体就业，全年新增城镇就业5万人以上，转移农村劳动力6万人以上。鼓励自主创业和自谋职业，有效增加群众财产性和经营性收入。完善分配机制，落实最低工资制度，健全职工工资正常增长机制和支付保障机制，促进职工特别是一线职工工资收入提高。

　　健全社会保障体系。完善社会救助和保障标准与物价上涨挂钩联动机制，切实保障困难群众的基本生活。创新征地补偿和失地农民安置、保障机制，做好城区回迁户安置工作。提高社会保险覆盖面和企业退休人员养老金水平，完善医疗、工伤、生育、失业保险市级统筹，确保各项待遇按时足额发放。继续推进城乡居民社会养老保险工作，新增养老保险13.6万人。加强保障性安居工程建设，新建保障性住房1.6万套，完善保障性住房准入机制。重视发展社会福利、红十字、慈善和残疾人事业，出台对贫困家庭精神病患者的救助制度。加大老年人活动场所设施建设力度，提倡居家养老，建立健全社会养老服务体系。加强妇女儿童工作，保护好妇女儿童合法权益。

　　加快发展社会事业。继续办好一批为民办实事项目。深入实施学前教育发展三年行动计划，新改扩建50所公办幼儿园。全面完成173万平方米中小学校安工程重建任务，实施农村义务教育阶段寄宿生营养改善工程。提高高中教育质量。加大校园及周边治安综合治理力度，深入开展校车安全整治。支持中高职院校改革发展，培养更多适用人才。加强各级各类教育教师队伍建设。重视发展特殊教育，广泛开展社区教育，构建终身教育体系。提升卫生服务能力。开工建设市妇幼保健医院，新建7所专科医院，续建6个病房大楼、10个卫生监督所项目，配全16所社区卫生服务中心基本诊疗设备，完善468所村卫生所规范化建设，全市力争新增床位2570张，增长25%。畅通人才引进机制，力争招聘各类医疗卫生人才1000名，引进高层次卫生人才100名，培训全科医生和乡村医生4500名。健全公共卫生和传染病防控体系，加快公立医院综合改革步伐，强化药品监管，落实公共卫生服务项目，努力为群众提供安全、有效、方便、廉价的医疗卫生服务。全面加强人口和计划生育工作，稳定低生育水平，深入开展“创建幸福家庭，打造幸福漳州”活动。抓紧实施游泳、射击、重竞技等五个省运会体育场馆建设，加紧培养输送体育后备人才，促进竞技水平提升。加快有线电视数字化整体转换，提高电视网络服务水平。

　　加强和创新社会管理。深入开展“六五”普法活动，提高群众法律意识。完善信访工作制度，引导群众依法合理表达诉求。发挥维稳“三支队伍”作用，妥善化解和处置社会矛盾。推动社区矫正工作制度化。推进“平安漳州”建设，完善“党政领导、公安牵头、部门配合、社会参与”的社会治安防控体系。推行企业安全生产标准化建设，持续开展专项整治和隐患排查。加大力度治理“餐桌污染”，建设食品安全工程。加大网络信息监管力度。加强综合应急救援队伍建设。强化国防教育、国防动员、民兵预备役、海防、打私、人防工作，深化“双拥”共建活动，落实拥军优属政策。加强民族宗教工作，依法管理宗教事务。

　　（五）切实增强中心城市的辐射和带动功能

　　同城规划，用战略眼光设计城市。抓紧完善《厦漳泉大都市区同城化总体发展规划》和9个专项规划。确保厦漳跨海大桥通车、高速公路扩建完成，开工建设同城大道，推进城际轻轨、海底隧道前期工作，全面落实18个同城化启动项目，筛选确定第二批同城化启动项目，加快厦漳泉同城化步伐。制订出台《关于加强规划工作的若干意见》，推动规划工作全覆盖。按照“田园都市、生态之城”的目标定位，加紧修编《漳州市城市总体规划》，着眼做大做强中心市区，拉开城市框架，提高中心城市辐射带动能力。组织编制道路交通、水利水系、城市绿地、都市田园、重要节点等专项规划，做好圆山新城、新行政服务中心等重点片区的规划设计。

　　彰显个性，用精品标准建设城市。启动“城市建设五年行动计划”，实施城市建设“十大工程”，加快碧湖生态园、漳州体育场、奥林匹克体育公园等重大项目建设，打造城市新地标，凸显城市新亮点。加快建设以“四横、六纵、两桥”为主干的城市路网，构建便捷的交通网络体系。扎实抓好城乡环境“点、线、面”综合整治，筹划“五园一区”，规划建设城市郊野公园，加快城市片林建设，推进城市绿化花化美化，充分彰显“田园都市、生态之城”的城市个性和魅力。

　　创新机制，用资本理念经营城市。以城兴城、滚动发展，健全城市造血机制。巩固国家级卫生城市成果，深入推进网格化、数字化、精细化城市管理，探索部门联合执法、集中整治、全民参与的长效管理机制，积极创建全国文明城市。深化交通体系研究，加强道路交通组织，大力发展公共交通，规范车辆停放管理，新增停车位1000个以上。

　　（六）充分激发县域经济发展活力和动力

　　发挥优势、凸显特色。统筹推进城乡发展，引导各县（市、区）围绕市第十次党代会确定的发展定位，依托特色资源，发挥比较优势，强化规划引领，完善扶持政策，宜农则农、宜工则工、宜商则商，把优势产业做出特色，把特色产业做出规模，把规模产业做出品牌，每个县（市、区）集中突破1-2个重点优势产业，努力形成“一县一业”、“一镇一品”的发展格局，形成一批配套程度高、集聚效应强的产业集群。

激发活力、壮大民企。加大支持民营经济发展力度，用好国务院鼓励和引导民间投资的新36条政策以及配套措施，坚决清理、取消各种限制性规定，推动民营经济跨越发展。支持建设重大产业项目，鼓励开展兼并重组，加强民营企业上市辅导，推动5家以上企业上市，帮助拓展国内外市场，培育一批民营大企业大集团。鼓励引导民营企业发展战略性新兴产业，扶持发展科技型民营企业，推动企业管理精细化、产品品牌化、技术高端化，增强企业市场竞争力。

　　城乡一体、互动共进。按照“县城扩容提质、乡镇特色打造、农村整洁美化”的要求，深入实施“大城关”战略，完善县城总体规划和专项规划，加强县城基础设施建设，扩大路网框架，壮大县城体量。以东山县为示范点，加快推进城乡一体化进程。以4个省级试点镇、9个市级试点镇为示范，推进强镇扩权，完善配套政策，注重规划设计，加快建设一批各具特色的工业强镇、商贸重镇、旅游旺镇和历史文化名镇。加强农村住房建设管理，改变有新房无新村的状况，加快建设一批经济繁荣、环境优美、管理有序的中心村和新农村社区。实施新一轮扶贫开发和“造福工程”，落实3个省级重点县、37个重点村22.4万贫困人口扶贫开发任务，实施扶贫项目148个，建设20个百户搬迁示范点，搬迁偏僻自然村和地质灾害点群众2600户。

　　（七）全方位深层次推进改革扩大开放

　　深化漳台合作。深入实施《漳台产业深度对接工作方案》，积极承接台湾石油化工、电子信息、装备制造、文化创意等产业的整体转移，争取在引进台资大项目、好项目上有新突破。推动台商在漳设立独资医疗机构，开展电子商务合作，深化教育、科技、文化、旅游等领域交流合作。拓宽漳台人才交流合作领域，加大台湾高层次人才引进力度。加快文化生态园、海峡两岸林语堂文化博览园、漳浦乌石妈祖城等文化交流平台建设，深化族谱对接，扩大民间交流交往，推进乡镇对接，推动漳台基层互动合作。

　　拓展多元市场。鼓励企业参加各类重要展会，深度拓展自贸区等国际市场。深入开展“品牌兴企”活动，加快建设3个省级以上出口基地，培育壮大一批基地龙头企业和大型骨干出口企业，提高自主品牌和高附加值产品出口比重。用足用好外贸促进政策，积极争取设立保税物流园区等海关特殊监管区。加快电子口岸建设，支持企业参加各种国际认证，做好“两反两保”涉案企业的跟踪服务，确保外贸出口稳定增长。

　　推进各项改革。提高直接融资比重，有效利用债券、私募股权、信托、租赁以及BT、BOT等融资形式。深化国企改革，积极推动市属国有或国有控股企业嫁接央企，促进做大做强。积极推进县级国库集中支付管理改革。深化农村改革，促进农村土地承包经营权规范有序流转，推广政策性农业保险，发展壮大村集体经济，推进农村公益事业“一事一议”和农村债务化解工作，加强农村“六大员”队伍建设，增强农村发展活力。继续深化行政审批制度改革，加快行政服务中心标准化建设。加快推进统计制度改革，全力做好“企业一套表”工作。深入推进科技、教育、文化、医疗卫生体制改革和事业单位分类改革。

　　（八）下大力气推动文化大发展大繁荣

　　加强精神文明建设。扎实推进社会主义核心价值体系建设，大力弘扬“爱国爱乡、海纳百川、乐善好施、敢拼会赢”的福建精神。以“爱我漳州”为主题，以“创建文明城，迎接省运会”为主线，加强社会公德、职业道德、家庭美德、个人品德建设，着力深化文明村镇创建活动，在全社会形成积极向上的精神追求和健康文明的生活方式。开展“荣誉市民”评选授荣活动，进一步凝聚推进发展的强大合力。把诚信建设摆在突出位置，大力推进政务诚信、商务诚信、社会诚信和司法公信建设，在全社会广泛形成守信光荣、失信可耻的氛围。健全志愿服务机制，打造一批志愿服务活动品牌。

　　大力发展文化事业。加大公共财政对公益性文化事业的投入，完善市县两级“三馆”等文化基础设施，规划建设文化艺术中心和规划馆、博物馆，设立413个农家书屋，继续实施乡镇综合文化站改扩建工程和有线广播“村村响”工程，加快实施电影院建设工程，深入开展全民健身、全民阅读和科普活动，加强文化队伍建设。做好文化遗产保护传承和利用，推进“海丝”和“闽系红砖”申遗，加强历史街区、古村落等具有历史价值建筑的保护。扎实开展第二轮修志工作，积极发展文学、艺术、广播影视、新闻出版事业。

　　发展壮大文化产业。制定实施推动文化产业发展优惠政策。把文化产业作为调结构、转方式的重要引擎和新的经济增长点予以培育，加快九大文化项目建设，培育发展六大文化产业，努力构建地方特色鲜明、产业优势明显的文化产业发展格局。稳妥推进文艺院团体制改革，组建漳州演艺总公司和福建省艺术学院漳州分校。加快发展特色文化体验项目。加强文化执法队伍建设，促进文化市场健康发展。

　　（九）保护好青山绿水碧海蓝天

　　全面推进节能减排。按期完成国家和省下达的落后产能淘汰任务。整合提升造纸、皮革行业。构建生态补偿机制，完善差别化的区域开发配套政策，严把新上项目产业政策、资源消耗和环境保护关。加快城镇污水处理厂管网配套，完善雨污分流系统，提高城乡生活污水处理率。加强脱硫设施运行管理，强化污染减排能力建设，确保达标排放、有效减排。

强化环境综合整治。抓好重点流域水环境综合整治，严厉打击违法排污、滥采滥挖、非法捕捞等破坏生态环境行为，巩固提升九龙江流域漳州段水环境质量，确保县级以上集中式饮用水源水质稳定达标，解决农村60万人饮用水安全问题。加大“青山挂白”治理力度，完成对“沿江、沿路、环城”青山挂白点的恢复治理。实施农村环境连片整治，推行农村垃圾“村收集、镇转运、县处理”，建设一批农村环保示范工程。深化市区环境综合治理，集中整治城市内河、噪音和机动车尾气污染，严控重金属污染。强化环境安全隐患排查，增强突发环境事件应急处置能力。

加强生态保护建设。开展生态建设年活动，全面启动创建国家森林城市，深入开展林业三大功能体系和五大工程建设。强化饮用水源保护区、自然保护区、沿海防护林、生态公益林、水仙花原产地和荔枝海等生态功能区的强制性保护。严格执行国家土地政策，守住基本农田红线。运用土地价格机制，引导节约集约用地。加强矿产资源管理，严格河道采砂监管。实施“碧海银滩”工程，加强海洋自然保护区和海洋生态功能区建设，构建海洋生态文明示范区。

　　四、切实加强政府自身建设

　　建设创业创新、又富又美的新漳州对政府工作提出了新的更高要求。各级政府及其工作部门要以转变职能为核心，以提高执行力为重点，努力建设学习型、服务型、效能型、法治型、廉洁型政府。

坚持科学理政，建设学习型政府。树立终身学习的理念，进一步提高学习力，真正做到学以立德、学以增智、学以创业，不断提高推动科学发展、跨越发展的能力和水平。树立世界眼光，培养战略思维，不断提高应对复杂局面和解决实际问题的能力和水平。

　　坚持为民执政，建设服务型政府。树立正确政绩观，始终把人民放在心中最高位置，以敬畏之心、感恩之心对待群众，重大决策顺民而定，工作措施应民而出，发展成果全民共享，着力解决群众最关心的热点问题、最直接的生计问题、最现实的利益问题。加强“12345”政务服务热线平台建设，更好地倾听民意、集中民智、化解民忧。

　　坚持高效施政，建设效能型政府。讲求实际，注重实效，不搞短期行为，不做表面文章。把心思放在谋发展上，把精力花在干事业上，把功夫用在抓落实上。对看准的事、议定的事，立说立行、一抓到底，确保部署落实到位。强化机关效能建设，全面落实绩效管理，完善跟踪督查和责任追究制度，严格考核评估奖惩，着力治庸治懒治散，提高行政效率。

　　坚持依法行政，建设法治型政府。把依法行政贯穿到决策、执行、监督的全过程，规范行政行为，做到用制度管权、按制度办事、靠制度管人。自觉接受人大法律监督、工作监督和政协民主监督，主动接受新闻舆论和社会公众监督。组建政府决策咨询顾问机构，建立健全先调研后决策的工作机制，提高决策科学化水平。

　　坚持从严治政，建设廉洁型政府。严格落实党风廉政建设责任制，持续推进惩治和预防腐败体系建设。加强对项目招投标、经营性土地招拍挂、产权交易、政府采购等重点领域的管理和监督，进一步明晰政府、市场和社会边界，压缩行政裁量空间，推进权力运行程序化、规范化和透明化。加强对公务员的教育、管理和监督，筑牢拒腐防变的思想道德防线，努力建设一支人民满意的公务员队伍。

　　各位代表，新的形势催人奋进，新的目标鼓舞人心，新的任务光荣而艰巨。让我们更加紧密地团结在以胡锦涛同志为总书记的党中央周围，在省委、省政府和市委的坚强领导下，团结和依靠全市人民，凝心聚力，开拓进取，为在更高起点上加快建设创业创新、又富又美的新漳州而努力奋斗，以优异的成绩迎接党的十八大胜利召开！
